# Effects of Polymorphisms in Myc-Related Genes on Bleeding Complications in Patients with Stable Warfarin Responses

**DOI:** 10.1155/2019/1813747

**Published:** 2019-05-08

**Authors:** Jeong Yee, Woorim Kim, Byung Chul Chang, Jee Eun Chung, Kyung Eun Lee, Hye Sun Gwak

**Affiliations:** ^1^College of Pharmacy & Division of Life and Pharmaceutical Sciences, Ewha Womans University, 52 Ewhayeodae-gil, Seodaemun-gu, Seoul 03760, Republic of Korea; ^2^College of Pharmacy, Chungbuk National University, 660-1, Yeonje-ri, Osong-eup, Heungdeok-gu, Cheongju-si 28160, Republic of Korea; ^3^Department of Thoracic and Cardiovascular Surgery, Bundang CHA Medical Center, CHA University, 59 Yatap-ro, Bundang-gu, Seongnam, Gyeonggi-do, Republic of Korea; ^4^Department of Thoracic & Cardiovascular Surgery, Yonsei University Medical Center, 50-1 Yonsei-ro, Seodaemun-gu, Seoul 03722, Republic of Korea; ^5^College of Pharmacy, Hanyang University, 55 Hanyangdeahak-ro, Sangnok-gu, Ansan 15588, Republic of Korea

## Abstract

**Objectives:**

This study aimed to identify the possible effects of* Myc* and 8q24 polymorphisms on bleeding complications in patients who maintained international normalized ratio (INR) of 2.0-3.0 with warfarin therapy after cardiac valve replacement.

**Methods:**

Twenty-five single nucleotide polymorphisms were analyzed, including* VKORC1*,* CYP2C9*,* Myc*, and 8q24. Univariate and multivariate analyses were conducted to evaluate the associations between genetic polymorphisms and bleeding complications. Attributable risk and the number needed to genotype (NNG) were also calculated to evaluate the potential clinical value of genotyping.

**Results:**

We included 142 patients, among whom 21 experienced bleeding complications. Multivariate models showed that patients carrying the CC genotype of rs6983561 and the A allele of rs13281615 at 8q24 had 27.6- and 10.0-fold higher bleeding complications, compared with patients with the A allele and the GG genotype, respectively. For rs6983561, the attributable risk and NNG were 96.4% and 36.8, respectively, whereas, for rs13281615, the attributable risk and NNG were 90.0% and 8.3, respectively. Atrial fibrillation was associated with a 5.5-fold increased risk of bleeding complications. The AUROC value was 0.761 (95% CI 0.659-0.863, p<0.001), and the Hosmer–Lemeshow test showed that the fitness of the multivariate analysis model was satisfactory (*χ*^2^=0.846; 3 degrees of freedom; p=0.838).

**Conclusions:**

Bleeding complications during warfarin therapy were associated with 8q24 polymorphisms and atrial fibrillation in patients with mechanical heart valves.

## 1. Introduction

Warfarin is a widely used anticoagulant, and its effectiveness is well established, mainly for preventing and treating atrial fibrillation, ischemic stroke, deep vein thrombosis, and pulmonary embolism [1, 2]. Although an introduction of direct oral anticoagulants has attracted many patients, warfarin still remains the first-line anticoagulant for patients with heart valve prostheses [3]. However, warfarin has several shortcomings, such as a narrow therapeutic range and wide inter- and intra-individual variability. Hence, warfarin administration requires close monitoring, using the international normalized ratio (INR) [4].

The most common adverse effect of warfarin is bleeding. Even though an elevated INR is correlated with an increased warfarin-associated bleeding risk, bleeding complications also can be seen in patients at a therapeutic INR [5]. Some studies have demonstrated that—in addition to a high INR—age, hypertension, and concomitant aspirin use were patient-related risk factors for bleeding complications [5, 6]. Although Myc has potential roles in vascular regulation, to our knowledge, no study has yet investigated the association between* Myc* gene polymorphisms and bleeding complications in patients receiving warfarin.

Myc is known as a transcription factor of many different genes, and its predominant role is to increase the production of transcripts from active genes [7]. Among many functions, Myc is involved in angiogenesis-related gene expression in a cell-dependent manner [8]. Polymorphisms of* Myc* have been shown to result in vascular malformation [8], thereby increasing bleeding risks [9]. In this context, polymorphisms of* Myc* could induce bleeding complications, especially in patients at risk of bleeding, such as those receiving warfarin treatment.

Chromosome 8q24 is enhancer and physically interacts with* Myc* [10]. Since 8q24 has roles in* Myc* promoter activity, polymorphisms of 8q24 could affect Myc functions, including those related to vascular malformation [11]. Therefore, it is reasonable to speculate that polymorphisms of 8q24 could lead to bleeding complications. Although patients undergoing warfarin therapy are vulnerable to bleeding complications, there is no study on the association between* Myc* or 8q24 gene polymorphisms and bleeding in patients on warfarin.

Therefore, this study aimed to investigate the association between Myc-associated polymorphisms and the risk of bleeding complications in patients who maintained an INR between 2.0 and 3.0 with warfarin therapy after cardiac valve replacement.

## 2. Materials and Methods

### 2.1. Study Patients and Data Collection

Study patients were included from the Ewha-Severance Treatment (EAST) Group of Warfarin, which consists of 229 patients who received warfarin after mechanical heart valve replacement between January 1982 and December 2009 at Severance Cardiovascular Hospital of Yonsei University College of Medicine. Patients who maintained a stable INR (INR between 2.0 and 3.0 for at least three consecutive measurements) were eligible for the study. We excluded patients who experienced bleeding complications under conditions of supra- or subtherapeutic INR. Patients were also excluded if their complications were not verified by health professionals.

Patients were followed up continuously at the outpatient clinic of Severance Cardiovascular Hospital of Yonsei University Medical Center. Blood samples were collected during regularly scheduled clinic visits. Data collection was performed retrospectively from scanned medical records and electronic medical records of patient visits that occurred between June 1983 and August 2010. Data including gender, age, body weight, height, position of valve prosthesis, valve type, warfarin therapy duration, INR measurements, concurrent medication, comorbidities, and history of bleeding complications were collected. Bleeding complications were classified as major life-threatening, other major, any major, minor, or minimal using the scheme detailed in the Platelet Inhibition and Patient Outcomes (PLATO) trial [12].

This study was approved by the Institutional Review Board of the Yonsei University Medical Center (approval number: 4-2009-0283). All patients gave written informed consent for participation.

### 2.2. Genotyping Methods

To select single nucleotide polymorphisms (SNPs) of* Myc* and 8q24 that might be associated with warfarin-related bleeding, genetic information concerning* Myc* and 8q24 was obtained from the PharmGKB database, Haploreg 4.1, and the National Center for Biotechnology (NCBI) SNP Database (dbSNP), as well as previous studies [10, 13–18]. Five* Myc* SNPs (rs4645957, rs4645948, rs4645962, rs4645943, and rs4645974) and 18 8q24 SNPs (rs6983267, rs1447295, rs4242382, rs4242384, rs7837688, rs16902094, rs4451114, rs1456315, rs6983561, rs16901979, rs10505483, rs13252298, rs1016343, rs10505477, rs9642880, rs13281615, rs1562430, and rs7014346) were selected. In addition to the selected SNPs,* VKORC1* rs9934438 and* CYP2C9* rs1057910, which were found to have significant effects on stable doses of warfarin, were also included in the study. Therefore, a total of 25 SNPs was investigated.

Genomic DNA from the patients was isolated from ethylenediaminetetraacetic acid-blood samples using the QIAamp DNA Blood Mini Kit (QIAGEN GmbH, Hilden, Germany) according to the manufacturer's instructions. Genotyping was carried out using a single-base primer extension assay and SNaPShot multiplex kits (ABI, Foster City, CA, USA) or the TaqMan genotyping assay with a real-time polymerase chain reaction (PCR) system (ABI 7300, ABI, Foster City, CA, USA), according to the manufacturer's instructions.

### 2.3. Statistical Analysis

Continuous variables in patients with bleeding complications and those without complications were compared using Student's t-test. Chi-square analysis was used to compare categorical variables between the two groups. Multivariate logistic regression analysis was used to examine the independent risk factors for bleeding complications. Factors having* p* values less than 0.05 from univariate analysis along with clinically relevant confounders were included in the multivariate analysis. Odds ratio (OR) and adjusted odds ratio (AOR) were calculated from the univariate and multivariate analyses, respectively. Attributable risk (%) was calculated using the formula (1-1/AOR) × 100. To test the model's goodness of fit, we performed a Hosmer–Lemeshow test. Discrimination of the model was further assessed by an analysis of the area under the receiver operating curve (AUROC), which assesses the ability of the risk factor to predict bleeding. We calculated the number needed to genotype (NNG) for preventing one patient from experiencing a significantly higher incidence of bleeding complications by 1/absolute risk reduction. Absolute risk reduction was calculated by multiplying the relative risk reduction via genotyping by the risk of higher incidence of bleeding complications without genotyping. A* p* value of less than 0.05 was considered statistically significant. All statistical analyses were carried out using IBM SPSS Statistics, version 20 software (International Business Machines Corp., New York, USA).

## 3. Results

Of 229 patients in the EAST Group of Warfarin, 87 patients were excluded due to the following reasons: 28 patients did not reach stable INR, 4 patients had bleeding complications at supratherapeutic INR, and 55 patients had reportedly minimal bleeding complications that were not verified by health professionals. Accordingly, data from 142 patients who underwent cardiac valve replacement were included in the analysis.

The median age of included patients was 60 years (range 34–81 years). There were 52 (36.6%) males. The follow-up periods ranged from 1.0 to 29.7 years. The mean INR monitoring interval was 2.9 months, and the average number of INR measurements per patient was 23. As shown in Table 1, 21 patients (14.8%) had bleeding complications at therapeutic INR. Among them, 11 patients and 10 patients experienced minor bleeding and minimal bleeding complications, respectively. There were 31 bleeding episodes in 21 patients during study period. No significant difference was found between the two groups except for atrial fibrillation. Patients with atrial fibrillation had more bleeding complications at therapeutic INR than patients without atrial fibrillation (p=0.045).

As shown in Table 2, statistically significant associations between genotypes and bleeding complications were found for rs6983561, rs16901979, rs10505483, and rs13281615 of 8q24. There was linkage disequilibrium (LD) among rs6983561, rs16901979, and rs10505483 (r^2^ >0.95). Patients with the CC genotype in rs6983561 (AA genotype in rs16901979 and TT genotype in 10505483) experienced more bleeding complications than A allele (C allele in rs16901979 and rs10505483) carriers (44.4% vs 12.8%, p=0.010). For rs13281615, patients with the A allele showed higher bleeding risks, compared with those with variant-type homozygote (19.0% vs 4.8%, p=0.029).

The multivariate analysis (Table 3) included sex, age, and factors having p<0.05 from the univariate analysis, including atrial fibrillation, rs6983561, and rs13281615 of 8q24. Because significant LD was observed between rs6983561, rs16901979, and rs10505483, only rs6983561 was included in the multivariate analysis. Among included factors, atrial fibrillation, rs6983561, and rs13281615 were significantly associated with bleeding complications (p=0.021, p=0.001, and p=0.019, respectively). After adjusting for related covariates, CC genotype carriers in rs6983561 showed about a 27.6-fold higher bleeding complication rate than A allele carriers. For rs13281615, A allele carriers had about a 10.0-fold higher bleeding complication rate than those with the GG genotype. Patients with atrial fibrillation had a 5.5-fold higher bleeding complication rate at a therapeutic INR than those without atrial fibrillation. The NNG for preventing one patient with the CC genotype in rs6983561 from suffering a higher incidence of bleeding complications was 36.8, and the NNG for rs13281615 was 8.3.

**Figure 1:**
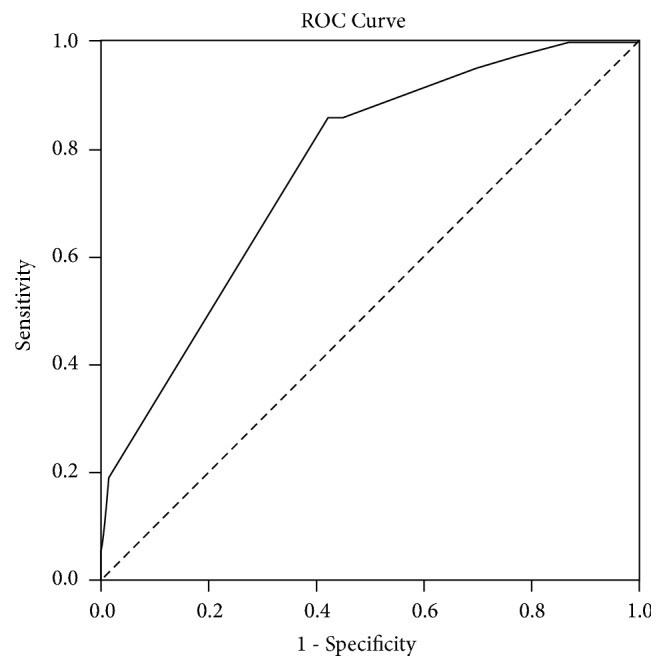
Area under receiver operating characteristic curve for bleeding complications at a therapeutic INR. Model included sex, age, atrial fibrillation, rs6983561, and rs13281615 for analysis. AUC is 0.761 (95% CI 0.659-0.863, p<0.001).

The AUROC value was 0.761 (95% confidence interval [CI] 0.659-0.863, p<0.001) (Figure 1). The Hosmer–Lemeshow test showed that the fitness of the multivariate analysis model was satisfactory (*χ*^2^=0.846; 3 degrees of freedom; p=0.838).

## 4. Discussion

The results of this study suggested that two SNPs of 8q24 (rs6983561 and rs13281615) were associated with bleeding complications at a therapeutic INR during warfarin treatment for mechanical heart valve patients. CC genotype carriers of rs6983561 were 27.6 times (95% CI 3.6-210.3) more likely to experience bleeding complications, compared with A allele carriers, and the attributable risk was 96.4%. After adjusting for related covariates, A allele carriers in rs13281615 were about 10.0 times more likely to experience bleeding complications than those with the GG genotype (95% CI 1.5-68.6); the attributable risk was 90.0%. Among comorbidities, atrial fibrillation was the only significant risk factor for bleeding complications after adjusting for covariates. The AUROC value of the multivariate analysis model for predicting bleeding complications was 0.76.

*Myc*, which is located on chromosome 8q24, is a transcription factor regulating 10-15% of all cellular genes. It is involved in cell progression, differentiation, apoptosis, and neoplasia [19]. It is widely known as a protooncogene; however, a previous study has found that it is also related to cardiovascular diseases [20]. Myc is reported to have critical roles in angiogenesis, and it has been suggested that it coordinates the angiogenic network as a master regulator [21]. The proper expression of Myc has been shown to be essential for vessel formation and smooth muscle cell proliferation [8, 22].

Hemostasis is a multiphase process involving blood vessels, platelets, and coagulation factors; an imbalance in any of the steps of hemostasis may result in bleeding [23]. Vascular malformation with unrestrained angiogenesis is among the causes of bleedings in several organs (e.g., gastrointestinal tract, retina, and endometrium) [24–26]. As Myc is involved in vascular formation and regulation, polymorphisms of the* Myc* gene may lead to vascular malformation, thereby increasing the risk of bleeding complications, especially among patients taking warfarin.

Chromosome 8q24 is one of the gene deserts; however, it has been suggested that the 8q24 region has regulatory elements for the expression of Myc. It is shown that polymorphisms of 8q24 interact near the* Myc* promoter region and alter Myc expression [11, 27]. In genome-wide association studies, 8q24 polymorphisms were also identified as cancer-related variant. As* Myc* is widely known as protooncogene, the effect of 8q24 polymorphisms was explained to be Myc-mediated in those studies [27, 28].

Rs6983561, along with its LD correlates (rs16901979 and rs10505483), is one of the SNPs that were significantly associated with bleeding complications in our study. It is located in the most centromeric region of 8q24, called region 2 (128.14-128.28 Mb). Some polymorphisms in region 2, including rs6983561 and rs16901979, have consistently shown strong associations with prostate cancer. It was explained that the polymorphisms possibly affect Myc expression, thereby increasing cancer risk [29, 30]. Our result showed that cancer-risk variants also increased bleeding complications, implying that bleeding is caused by alterations of Myc expression.

Rs13281615 is known to be associated with breast cancer [31]. Interestingly, this variant allele, which is reported to increase breast cancer risk, showed a protective effect against bleeding complications in our study. Some studies have suggested that this SNP does not involve a cis-regulatory element for Myc expression and alters plasmacytoma variant translocation 1 (PVT1) expression, indicating that this SNP might be involved in different mechanisms for bleeding from those of other variants [28, 31].

In our study,* VKORC1* and* CYP2C9* polymorphisms were not significantly different in terms of bleeding complications. Although VKORC1 and CYP2C9 are known to affect warfarin dose [2], our study patients, who already had dosing adjustments according to INR measurements, were expected not to be affected by* VKORC1* and* CYP2C9* polymorphisms.

We found that atrial fibrillation was a risk factor for bleeding complications. A study has reported that atrial fibrillation increases the bleeding risk [32]. However, there is also conflicting report about the impact of atrial fibrillation on bleeding complications [33]. Further research needs to be performed to confirm the association between atrial fibrillation and bleeding.

The NNG for preventing bleeding complications in patients with high-risk genotypes was calculated to show the clinical significance and cost-effectiveness of genotyping. The NNGs of rs6983561 and rs13281615 were 36.8 and 8.3, respectively, which implies that genotyping for these SNPs before treatment might be helpful for reducing the risk of bleeding complications associated with warfarin.

Although this study was limited by its retrospective design and small sample size, to our knowledge this was the first study to investigate the association between Myc-related genes—including 8q24 polymorphisms—and warfarin-related bleeding complications. Moreover, we calculated the attributable risks and NNGs of each high-risk SNP, which can be used to individualize warfarin therapy. Since this study dealt with patients with INR 2-3, only minimal or minor bleeding events were observed. While there is no doubt that fatal and major hemorrhages are of essential importance, minor bleedings are also important, since they serve as an alert for subsequent major bleedings and may increase the number of visits to clinics and sometimes the emergency room (ER), which results in additional expenditures. They also can result in permanent withdrawal of warfarin therapy, thus depriving patients of the effective therapy available.

## 5. Conclusions

This study showed that two SNPs of 8q24 (rs6983561 and rs13281615) were associated with bleeding complications at a therapeutic INR during warfarin treatment for mechanical heart valve patients. Given the retrospective study design and the relatively small sample size, our hypothesis requires further independent validation using prospective study design with large sample size.

## Figures and Tables

**Table 1:** Patient characteristics of study patients.

Characteristics	Bleeding complication, number (%)		*p*
	Presence (n = 21)	Absence (n = 121)	
Sex			0.705
Male	8 (38.1)	44 (36.4)	
Female	13 (61.9)	77 (63.6)	
Age (years)			0.106
Mean ± SD	62.0 ± 11.2	58.7 ± 10.0	
Body weight (kg)			0.989
Mean ± SD	58.6 ± 10.7	58.7 ± 10.4	
Body mass index (kg/m^2^)			0.756
Mean ± SD	22.3 ± 2.3	22.5 ± 2.8	
Comorbidity			
Hypertension	6 (28.6)	33 (27.3)	0.902
Diabetes mellitus	3 (14.3)	10 (8.3)	0.377
Chronic heart failure	7 (33.3)	25 (20.7)	0.199
Atrial fibrillation	17 (81)	70 (57.9)	0.045
Myocardial infarction	2 (9.5)	2 (1.7)	0.104
Comedication			
Angiotensin-converting-enzyme inhibitor	2 (10.5)	19 (18.8)	0.383
Angiotensin II receptor blocker	4 (21.1)	19 (18.8)	0.820
Antiplatelet drugs	0 (0)	4 (3.8)	0.398
Calcium channel blocker	4 (21.1)	19 (18.8)	0.820
Diuretics	9 (47.4)	35 (34.7)	0.291
Statins	0 (0)	4 (4.0)	0.378
Valve position			0.740
Aortic	6 (28.6)	28 (23.1)	
Mitral	9 (42.9)	66 (54.5)	
Double^a^	5 (23.8)	20 (16.5)	
Tricuspid^b^	1 (4.8)	7 (5.8)	
Valve type			0.418
St. Jude Medical	7 (38.9)	39 (34.2)	
CarboMedics	6 (33.3)	32 (28.1)	
ATS	2 (11.1)	15 (13.2)	
MIRA	1 (5.6)	9 (7.9)	
Duromedics	2 (11.1)	6 (5.3)	
OnX	0 (0)	4 (3.5)	
Others^c^	0 (0)	9 (7.9)	
International normalized ratio			0.143
Mean ± SD	2.41 ± 0.07	2.45 ± 0.10	
Follow-up time (years)			0.886
Median (range)	14.3 (1.4 - 29.7)	14.7 (1.0 – 27.7)	

^a^Aortic plus mitral valve; ^b^tricuspid valve with or without other valves; ^c^including Sorin, Bjork Shiley, D-ring, and prostheses using two or more different valve types.

**Table 2:** Factors associated with bleeding complications at a therapeutic INR.

Gene polymorphism	Allele change	Minor allele frequency	Grouped genotypes	Bleeding complications, number (%)		OR (95% CI)	*P*
				Presence (n = 21)	Absence (n = 121)		
*VKORC1 *	C>T	0.113	CC, CT	3 (14.3)	27 (22.3)	1	0.405
rs9934438			TT	18 (85.7)	94 (77.7)	1.72 (0.47-6.29)	
*CYP2C9 *	A>C	0.043	AA	18 (85.7)	111 (92.5)	1	0.304
rs1057910^a^			AC, CC	3 (14.3)	9 (7.5)	2.06 (0.51-8.32)	
8q24	G>T	0.384	GG	4 (19.0)	16 (13.2)	1	0.479
rs6983267			GT, TT	17 (81.0)	105 (86.8)	0.65 (0.19-2.17)	
8q24	A>C	0.162	AA	0 (0.0)	2 (1.7)	1	0.553
rs1447295			AC, CC	21 (100.0)	119 (98.3)	0.98 (0.96-1.01)^*∗*^	
8q24	A>G	0.162	AA	0 (0.0)	5 (4.1)	1	0.343
rs4242382			AG, GG	21 (100.0)	116 (95.9)	0.96 (0.92-1.00)^*∗*^	
8q24	C>A	0.155	CC	0 (0.0)	4 (3.3)	1	0.398
rs4242384			CA, AA	21 (100.)	117 (96.7)	0.97 (0.94-1.00)^*∗*^	
8q24	T>G	0.141	TT	0 (0.0)	5 (4.1)	1	0.343
rs7837688			TG, GG	21 (100.0)	116 (95.9)	0.96 (0.92-1.00)^*∗*^	
8q24	A>G	0.278	AA, AG	18 (85.7)	113 (93.4)	1	0.225
rs16902094			GG	3 (14.3)	8 (6.6)	2.35 (0.57-9.71)	
8q24	T>C	0.454	TT, TC	16 (76.2)	99 (81.8)	1	0.544
rs445114			CC	5 (23.8)	22 (18.2)	1.41 (0.47-4.25)	
8q24	T>C	0.304	TT, TC	19 (95.0)	105 (87.5)	1	0.329
rs1456315			CC	1 (5.0)	15 (12.5)	0.37 (0.05-2.96)	
8q24	A>C	0.254	AA, AC	17 (81.0)	116 (95.9)	1	0.010
rs6983561			CC	4 (19.0)	5 (4.1)	5.45 (1.33-22.36)	
8q24	C>A	0.261	CC, CA	17 (81.0)	116 (95.9)	1	0.010
rs16901979			AA	4 (19.0)	5 (4.1)	5.46 (1.33-22.36)	
8q24	C>T	0.261	CC, CT	17 (81.0)	116 (95.9)	1	0.010
rs10505483			TT	4 (19.0)	5 (4.1)	5.46 (1.33-22.36)	
8q24	A>G	0.292	AA, AG	17 (81.0)	106 (87.6)	1	0.409
rs13252298			GG	4 (19.0)	15 (12.4)	1.66 (0.49-5.61)	
8q24	C>T	0.340	CC, CT	17 (81.0)	107 (89.2)	1	0.286
rs1016343			TT	4 (19.0)	13 (10.8)	1.94 (0.56-6.64)	
8q24	A>G	0.391	AA, AG	12 (57.1)	77 (63.6)	1	0.570
rs10505477			GG	9 (42.9)	44 (36.4)	1.31 (0.51-3.36)	
8q24	G>T	0.261	GG, GT	17 (81.0)	112 (92.6)	1	0.089
rs9642880			TT	4 (19.0)	9 (7.4)	2.93 (0.81-10.57)	
8q24	A>G	0.465	AA, AG	19 (90.5)	81 (66.9)	1	0.029
rs13281615			GG	2 (9.5)	40 (33.1)	0.21 (0.05-0.96)	
8q24	T>C	0.170	TT, TC	21 (100.0)	113 (94.2)	1	0.256
rs1562430			CC	0 (0.0)	7 (5.8)	0.94 (0.90-0.98)^*∗*^	
8q24	A>G	0.278	AA	3 (14.3)	7 (5.8)	1	0.160
rs7014346			AG, GG	18 (85.7)	114 (94.2)	0.37 (0.09-1.57)	
*Myc*	C>T	0.113	CC, CT	21 (100.0)	120 (99.2)	1	0.676
rs4645957			TT	0 (0)	1 (0.8)	0.99 (0.98-1.01)^*∗*^	
*Myc*	C>T	0.150	CC, CT	20 (95.2)	117 (98.3)	1	0.369
rs4645948			TT	1 (4.8)	2 (1.7)	2.93 (0.25-33.79)	
*Myc*	T>C	0.011	TT	21 (100.0)	118 (97.5)	1	0.466
rs4645962			TC, CC	0 (0.0)	3 (2.5)	0.98 (0.95-1.01)^*∗*^	
*Myc*	C>T	0.302	CC	8 (42.1)	55 (49.1)	1	0.572
rs4645943			CT, TT	11 (57.9)	57 (50.9)	1.33 (0.50-3.55)	
*Myc*	C>T	0.159	CC	11 (61.1)	84 (73.7)	1	0.270
rs4645974			CT, TT	7 (38.9)	30 (26.3)	1.78 (0.63-5.02)	

*∗*Data are expressed as relative risks.

^a^Patients with the CC genotype were not found in this study.

**Table 3:** Multivariate analysis to identify predictors for bleeding complications at a therapeutic INR.

Variables	Adjusted OR (95% CI)	Attributable risk (%)	NNG^a^
Atrial fibrillation	5.50 (1.30-23.36)^*∗*^	81.83	
8q24 rs6983561, CC	27.64 (3.63-210.29)^*∗∗*^	96.38	36.8
8q24 rs13281615, AA, AG	10.00 (1.47-68.6)^*∗*^	90.00	8.3

Logistic regression analyses were carried out with variables of sex, age, atrial fibrillation, rs6983561, and rs13281615.

^*∗*^ *p* < 0.05; ^*∗∗*^*p* < 0.01.

^a^Number needed to genotype (NNG) was calculated with the formula 1/(attributable risk × proportion of patients with both bleeding complications and risk genotypes).

## Data Availability

The data used to support the findings of this study are available from the corresponding author upon request.
